# Botulinum toxin type-A in the prophylactic treatment of medication-overuse headache: a multicenter, double-blind, randomized, placebo-controlled, parallel group study

**DOI:** 10.1007/s10194-011-0339-z

**Published:** 2011-04-16

**Authors:** Giorgio Sandrini, Armando Perrotta, Cristina Tassorelli, Paola Torelli, Filippo Brighina, Grazia Sances, Giuseppe Nappi

**Affiliations:** 1 Headache Science Center, IRCCS ‘‘C. Mondino Institute of Neurology” Foundation, Via Mondino 2, 27100 Pavia, Italy; 2 Headache Clinic, IRCCS Mediterranean Neurological Institute “Neuromed”, Pozzilli, Isernia Italy; 3 Headache Science Center, IRCCS ‘‘C. Mondino Institute of Neurology” Foundation, University of Pavia, Via Mondino 2, 27100 Pavia, Italy; 4 Headache Centre, Department of Neuroscience, University of Parma, Parma, Italy; 5 Department of Clinical Neurosciences, University of Palermo, Palermo, Italy

**Keywords:** Botulinum toxin type-A, Medication-overuse headache, Prophylactic treatment, Migraine, Pericranial muscle tenderness

## Abstract

Medication-overuse headache (MOH) represents a severely disabling condition, with a low response to prophylactic treatments. Recently, consistent evidences have emerged in favor of botulinum toxin type-A (onabotulinum toxin A) as prophylactic treatment in chronic migraine. In a 12-week double-blind, parallel group, placebo-controlled study, we tested the efficacy and safety of onabotulinum toxin A as prophylactic treatment for MOH. A total of 68 patients were randomized (1:1) to onabotulinum toxin A (*n* = 33) or placebo (*n* = 35) treatment and received 16 intramuscular injections. The primary efficacy end point was mean change from baseline in the frequency of headache days for the 28-day period ending with week 12. No significant differences between onabotulinum toxin A and placebo treatment were detected in the primary (headache days) end point (12.0 vs. 15.9; *p* = 0.81). A significant reduction was recorded in the secondary end point, mean acute pain drug consumption at 12 weeks in onabotulinum toxin A-treated patients when compared with those with placebo (12.1 vs. 18.0; *p* = 0.03). When we considered the subgroup of patients with pericranial muscle tenderness, we recorded a significant improvement in those treated with onabotulinum toxin A compared to placebo treated in both primary (headache days) and secondary end points (acute pain drug consumption, days with drug consumption), as well as in pain intensity and disability measures (HIT-6 and MIDAS) at 12 weeks. Onabotulinum toxin A was safe and well tolerated, with few treatment-related adverse events. Few subjects discontinued due to adverse events. Our data identified the presence of pericranial muscle tenderness as predictor of response to onabotulinum toxin A in patients with complicated form of migraine such as MOH, the presence of pericranial muscle tenderness and support it as prophylactic treatment in these patients.

**Electronic supplementary material:**

The online version of this article (doi:10.1007/s10194-011-0339-z) contains supplementary material, which is available to authorized users.

## Introduction

The 2nd edition of the International Headache Society’s International Classification of Headache Disorders (ICHD-II, IHS 2004) [1] introduced the term medication-overuse headache (MOH: code 8.2, ICHD-II) to indicate a chronic daily headache condition in which an excessive intake of symptomatic drugs has played a role in the chronification, and in which a clear relationship between increased drug intake and worsening of the headache is detectable [2]. The prevalence of MOH ranges from 1 to 5% in the general population [3, 4], rising to 10% in headache clinic patients [5] and to 80% among patients with chronic migraine in a tertiary headache center population [6]. MOH represents a severely disabling condition affecting social life and work ability, with a low response to prophylactic treatments in the absence of a concomitant drug withdrawal treatment (see [7] for review), but also with a very high incidence of relapse (between 30 and 50% of patients) within the first year after withdrawal treatment [8].

Intramuscular injections of botulinum toxin type-A (onabotulinum toxin A) has been employed to treat headache pain including episodic migraine [9, 10] and chronic tension-type headache [11–13] without univocal results as prophylactic treatment and chronic daily headaches not responding to previous prophylactic treatments with encouraging results [14]. On the contrary, growing consistent evidences are emerging in favor of onabotulinum toxin A as prophylactic treatment in chronic migraine (1.5.1, ICHD-II) [15–17] and, in particular, in subgroups of patients with cutaneous allodynia, pericranial muscular tenderness [18] or specific types of headache pain such as the so defined “imploding” and “ocular” pain [19, 20].

To explain the prophylactic effect of onabotulinum toxin A, it has been hypothesized that this neurotoxin could prevent or reduce the abnormal peripheral sensory signals from the pericranial muscles to the central nervous system and/or inhibit the sensitization of nociceptive neurons in the dorsal horn [21]. As in chronic daily headache, including MOH patients with migraine as primary headache, both pericranial muscle tenderness [22] and sensitization of the pain pathways at the trigeminal [23] and spinal levels [24] has been demonstrated, one would predict that in these patients onabotulinum toxin A would further improve the benefit of the withdrawal treatment and so facilitate the reversion to an episodic form of headache.

Our study was aimed to evaluate in a multicenter, double-blind placebo-controlled study the efficacy and safety of onabotulinum toxin A as prophylactic treatment for patients with MOH with migraine as primary headache, as well as to address if specific features such as cephalic allodynia, pericranial muscle tenderness, type of headache pain or of drug overuse may influence the response to onabotulinum toxin A.

## Materials and methods

### Study design

**Fig. 1:**
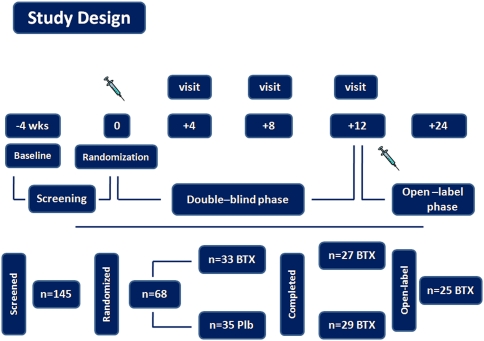
Study design

The enrollment phase was conducted from January 2006 to July 2008 at: The Headache Science Center, IRCCS C. Mondino Institute of Neurology Foundation, Pavia, Italy; Headache Center, University of Parma, Parma, Italy; Headache Center, University of Palermo, Palermo, Italy. The study had a 4-week baseline screening phase (referred to as baseline) and a 12-week double-blind, parallel group, placebo-controlled phase with one injection cycle at day “0” of the double-blind phase, followed by a 12-week, open-label phase (details will be described separately) (Fig. 1). All the potential participants were selected from among patients on the waiting list for a consultation in outpatient headache clinics of the participating center. All the patients enrolled in the study filled in a daily headache diary (mailed) to record their headache symptoms and acute treatments every day for at least 2 months before the start of the baseline period (4 weeks) and for the entire period of the study. The study was conducted in accordance with the Declaration of Helsinki Ethical Principles and Good Clinical Practices and was approved at each site by an independent local ethics committee. Written informed consent was obtained from each participant prior to any study-related procedures.

### Study population

Eligible patients were men or women aged 18–65 years with a history of headache, fulfilling the diagnostic criteria for migraine without aura (coded as 1.1) [1] as primary headache plus medication-overuse headache (coded as 8.2) [1, 2] with ≥15 headache days every 4 weeks in the past 3 months and with each headache day consisting of ≥4 h of continuous headache prevalent with migraine features.

Exclusion criteria were definite or suspected diagnosis of pathologies affecting neuromuscular function including, myasthenia gravis, Eaton–Lambert syndrome and amyotrophic lateral sclerosis, and presence of cervical pathologies or other factors liable to give rise to pericranial muscle disorders.

Further exclusion criteria included: other primary or secondary headaches, including a history of complicated migraine (i.e., migrainous infarction, hemiplegic migraine, basilar migraine or ophthalmoplegic migraine); any serious systemic or neurological diseases or psychiatric disorders, including depression (Beck’s Depression Inventory score >17 at day 1 of baseline); temporo-mandibular disorder, fibromyalgia, complex regional pain syndrome or neuropathic pain [25–27]; use of prophylactic medications for headaches, use of opiates, antidepressants, benzodiazepines, hormones, muscle relaxants and agents that may interfere with neuromuscular function within 4 weeks of day 1 of the baseline; previous exposure to any botulinum toxin serotype for other pathological conditions or for other purposes. Women of childbearing potential were required to have negative urine pregnancy test. Females who were pregnant, nursing or planning a pregnancy during the study, or who were unable or unwilling to use a reliable form of contraception during the study were excluded.

### Randomization, stratification and study treatment

At the end of the baseline period, patients meeting the inclusion/exclusion criteria were admitted as inpatients (ordinary hospitalization or day hospital) and treated with standard withdrawal therapy for detoxification for 8 ± 2 days [28].

Patients were stratified based on the type and frequency of acute drug overused during baseline, in order to balance their distribution within the two study groups. The project statistician created a randomized treatment allocation schedule using a computer random number generator. Both the patient and principal investigator, as well as the co-investigators who administered the treatment and assessed the safety and outcomes and the sponsor of the study, were blinded as to the identity of the randomized study medication. Blinding was maintained by having a designated pharmacist (the only person to have access to the randomization list), who provided the principal investigator or study co-ordinator with a vial containing the study medication labeled with the patient’s sequential identification number from the randomized allocation schedule. All patients remained double blinded until the last patient had completed the study. The blind code could be broken by the principal investigator only for safety concern.

During the second day of hospitalization, all patients were randomized (1:1) in a double-blind fashion to onabotulinum toxin A (BOTOX, Allergan Inc., Irvine, CA, USA) or placebo treatment and received 16 (8 on the right and 8 on the left) intramuscular injections in the following muscles: frontalis (2 injection points), corrugators (1 injection point), temporalis (1 injection point), cervical paraspinal (2 injection points) and trapezius (2 injection points) for a total of 100 U for patients. Intramuscular injections were administered using a sterile 30-gauge, 0.5 inch needle and 0.2 ml (saline dilution) of onabotulinum toxin A (5 U) or placebo at each site, except for the trapezius where we administered 0.4 ml of onabotulinum toxin A (10 U). Patients were discharged and reassessed after 4, 8 and 12 weeks as outpatients. At the end of the study, all the onabotulinum toxin A responder patients were offered entry into a 12-week open-label follow-up phase to receive a second injection cycle (Fig. 1). All patients, including those who were not willing to participate in the long-term study, were re-assessed in a follow-up visit at 12 months.

During the withdrawal treatment, to mitigate possible rebound effects, the patients received twice a day, intravenous infusion of a saline solution plus a vitamin complex (B12, folic acid, PP, C), glutatione 600 mg, alizapride 0.25 mg and clordemetildiazepam (0.25 mg for the first 3 days, then gradually reduced until withdrawal in 4 days). Breakthrough migraine attacks were treated with ketoprofene, 100 mg i.m., as a rescue medication.

Safety was assessed by reports of adverse events, physical and neurological examination and laboratory tests. After treatment at day 0, adverse events were recorded and documented with information regarding the date of onset, severity, duration, resolution date, relationship with study treatment, treatment required and outcome.

### Outcome measures

The primary efficacy end point was mean change from baseline in frequency of headache days for the 28-day period ending with week 12. A headache day was defined as a calendar day (00:00 to 23:59) when the patient reported not <4 h of headache. Secondary efficacy end points were mean change from baseline in acute headache pain medication intakes and in days with acute headache medication consumption at 4, 8 and 12 weeks after onabotulinum toxin A administration. The intensity of the headache pain was evaluated by a 0–10 numerical rating scale (NRS) score. The analysis included two assessments of disability measured by Headache Impact Test (HIT)-6 score and Migraine Disability Assessment Scale (MIDAS) administered at 4 and 12 week after onabotulinum toxin A administration. Patients were subdivided into subgroups based on the presence/absence of cephalic cutaneous allodynia measured by a prospective clinical questionnaire [29], pericranial muscle tenderness, assessed by palpation and type of migraine pain (exploding, imploding, ocular) [19] at baseline. Other efficacy analysis included the incidence of subjects with no less than 50% decrease from baseline in the frequency of headache days for the 28-day period ending with week 12 (primary end point) and during the whole observed period from 4 to 12 weeks.

### Statistical analysis

Data analysis was performed using non-parametric statistics. Ordinal measurements, including age, headache days, acute headache medication intake, days with drug consumption, pain intensity and disability scales (MIDAS and HIT-6), were compared between groups using Mann–Whitney test. Ordinal measurements before and after treatment were compared using ANOVA for repeated measures. For post hoc analysis of group mean differences, we used Student’s *t* test with Bonferroni correction. Nominal data were analyzed using χ^2^ test. The level of significance was set at 0.05. All statistics were calculated using the SPSS (16.0) program for Windows (SPSS, Chicago, IL, USA).

## Results

### Demographic and baseline characteristics

Of the 145 patients screened, 68 were randomized to onabotulinumtoxinA (*n* = 33) or placebo (*n* = 35). Twelve (17.7%) subjects discontinued prior to week 12, six (8.8%) randomized to onabotulinum toxin A and six (8.8%) to placebo, and 56 (82.3%) completed the study, 27 (48.2%) randomized to onabotulinum toxin A and 29 (51.7%) to placebo. As a consequence, the number of participants was small and represented a limitation of the present work. In subjects who dropped-out, discontinuation was due to being lost to follow-up (1 onabotulinum toxin A; 2 placebo); adverse events (2 onabotulinum toxin A; 0 placebo) or personal reasons (3 onabotulinum toxin A; 4 placebo). Demographic and clinical characteristics of the study population at baseline are reported in Table 1. Headache pain intensity and assessment of disability (MIDAS and HIT-6) at baseline were reported as Online Resource 1. The prevalence of cutaneous allodynia, pericranial muscle tenderness, type of migraine pain and drug overuse is summarized in Table 2. There were no between-group significant differences at baseline for demographic and clinical characteristics (Table 1), as well as for headache pain intensity and disability measurements (MIDAS and HIT-6) (Online Resource 1).

**Table 1:** Demographic and clinical characteristics of the study population at baseline and at 4, 8 and 12 weeks

	BoNTA (*n* = 27)	Placebo (*n* = 29)	*p* values
Mean age (years)	48.5 ± 9.2 (28–65)	49.0 ± 10.1 (28–64)	0.806
Female	21	24	
Duration (years)	19.7	20.3	
Mean headache days/28 days			
Baseline	24.2 ± 5.0 (14–30)	25.5 ± 5.6 (15–30)	0.209
4	16.6 ± 8.2 (0–30)	19.0 ± 9.6 (0–30)	0.234
8	14.7 ± 9.1 (1–30)	18.0 ± 9.5 (0–30)	0.212
12	12.0 ± 9.0 (4–30)	15.9 ± 9.5 (0–30)	0.081
Mean acute pain drug consumption/28 days			
Baseline	31.0 ± 12.7 (12–60)	34.7 ± 18.5 (12–90)	0.675
4	14.6 ± 12.8 (0–56)	19.6 ± 15.3 (0–60)	0.192
8	16.2 ± 14.3 (2–60)	19.0 ± 15.5 (0–60)	0.478
12	12.1 ± 14.6 (0–58)	18.0 ± 14.4 (0–90)	0.030
Mean days with acute pain drug consumption/28 days			
Baseline	22.7 ± 6.4 (12–30)	23.6 ± 6.6 (12–30)	0.587
4	12.0 ± 9.0 (0–30)	15.3 ± 10.1 (0–30)	0.240
8	12.1 ± 9.5 (1–30)	15.1 ± 10.2 (0–30)	0.256
12	10.7 ± 10.1 (2–30)	14.3 ± 9.1 (0–30)	0.085

**Table 2:** Headache characteristics of the study population at baseline

	BoNTA (*n* = 27)	Placebo (*n* = 29)
Headache characteristics		
Cutaneous allodynia	21 (77.8%)	25 (86.2%)
Pericranial muscle tenderness	14 (51.9%)	15 (51.7%)
Exploding pain	12 (44.4%)	10 (34.5%)
Imploding pain	13 (48.1%)	14 (48.3%)
Ocular pain	2 (7.4%)	5 (17.2%)
Drug overused		
Combination	5 (18.5%)	6 (20.7%)
Ergot	1 (3.7%)	1 (3.4%)
FANS	10 (37.0%)	13 (44.8%)
Triptans	11 (40.7%)	9 (31.0%)

### Outcome measures

#### Overall subjects

When the whole group of randomized subjects (onabotulinum toxin A and placebo) was considered, despite a clear tendency of onabotulinum toxin A-treated subjects to show better results than placebo-treated subjects, no significant differences were detected in primary (headache days) and secondary (acute pain drug consumption, days with acute pain drug consumption) end points, pain intensity and headache impact on functioning (HIT-6 and MIDAS), except for a significant reduction in mean acute pain drug consumption at 12 weeks in the onabotulinum toxin A-treated compared to placebo-treated patients (Table 1).

#### Subjects with pericranial muscle tenderness and cephalic allodynia

**Fig. 2:**
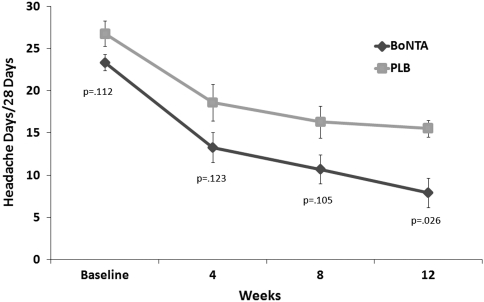
Primary end point: mean change (±SE) from baseline in frequency of headache days for the 28-day period in MOH patients with pericranial muscle tenderness

**Fig. 3:**
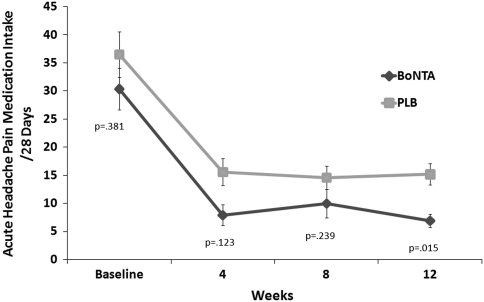
Secondary end point: mean change (±SE) from baseline in acute headache pain medication intake for the 28-day period in MOH patients with pericranial muscle tenderness

**Fig. 4:**
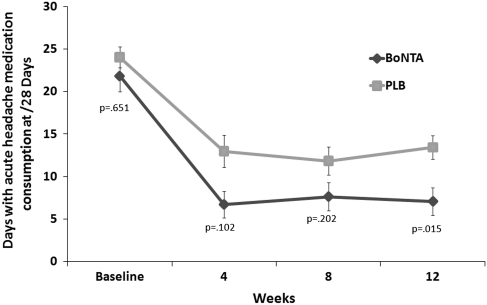
Secondary end point: mean change (±SE) from baseline in days with acute headache medication consumption in MOH patients with pericranial muscle tenderness

Significant differences in onabotulinum toxin A-treated versus placebo-treated patients were observed for primary and secondary end points at 12 weeks in those with pericranial muscle tenderness. In patients with pericranial muscle tenderness, onabotulinum toxin A-treatment was found to show, when compared with placebo treatment, a significant reduction in frequency of headache days (primary end point) (Fig. 2), as well as in both headache pain medication intake (Fig. 3) and days with acute headache medication consumption (Fig. 4) for the 28-day period at 12 weeks.

Furthermore, a statistical significant reduction in scores for pain intensity as well as for disability measures, MIDAS and HIT-6, were detected at both 4 and 12 weeks in onabotulinum toxin A-treated when compared with placebo-treated patients (Online Resource 2 Figs. 1–3).

No differences were detected at any time point between the onabotulinum toxin A- and placebo-treated subjects in primary and secondary end points, as well as in pain intensity and headache impact on functioning scores in subgroups of patients with cephalic allodynia (all *p* > 0.05).

#### Subjects with exploding versus imploding/ocular headache

In the subgroups of randomized subjects based on the type of headache pain (exploding and imploding/ocular), no significant differences were found between onabotulinum toxin A- and placebo-treated patients, except for a significantly better mean MIDAS score at 4 (*p* = 0.012) and 12 (*p* = 0.008) weeks in the exploding pain subgroup that had onabotulinum toxin A treatment when compared with placebo treatment.

#### Responders versus non-responders

**Fig. 5:**
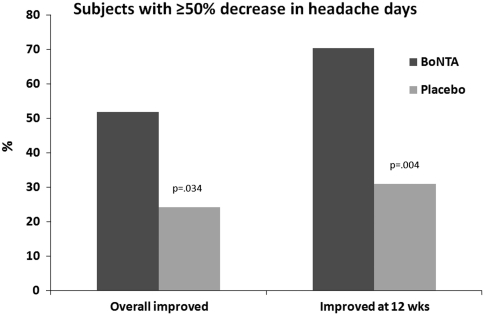
Percentage of patients with at least a 50% decrease from baseline in the frequency of headache days across all time points and at 12 weeks in BoNTA and placebo treated

A significantly greater percentage of onabotulinum toxin A-treated than placebo-treated patients had at least a 50% decrease from baseline in the frequency of headache days when both headache days at 12 weeks (*Z* = −2.915; *p* = 0.004) and that across all time points (*Z* = 2.121; *p* = 0.034) were considered (Fig. 5).

## Safety

A total of 16 (28.5%) subjects in the randomized population experienced adverse events. Treatment-related adverse events were reported in 25.9% of the onabotulinum toxin A-treated (7 patients) and in 17.2% of the placebo-treated (5 patients) patients. Two patients randomized to onabotulinum toxin A (7.4%) discontinued due to adverse events (neck pain). No clinically significant serious adverse events were reported in any of the 56 subjects. Most common adverse events (>5%) were pain at the site of injection and muscular weakness, all of which resolved without sequelae.

## Discussion

The study evaluated the efficacy and safety of onabotulinum toxin A as prophylactic treatment in MOH patients with migraine as primary headache, reporting on clinical features that could play as predictors of response to onabotulinum toxin A.

Results showed that when the whole group of randomized subjects was considered, a significant reduction in mean acute pain drug consumption at 12 weeks, as well as a clear but not significant trend toward better clinical results across all time points in primary and other secondary end points in favor of onabotulinum toxin A-treated when compared with placebo-treated patients was observed. It is worth noting that a significantly greater percentage of onabotulinum toxin A-treated with respect to placebo-treated patients showed 50% or more improvement in the primary end point (mean headache days), both when the 12-week time point and the entire post-treatment period, from 4 to 12 weeks, were considered.

The most relevant result of the study emerged when the subgroup of patients with muscular tenderness was considered. In this case, a significant improvement in both primary (mean headache days) and secondary end points (mean drugs consumption and mean days with consumption) at 12 weeks, as well as in pain intensity and headache impact on functioning (HIT-6 and MIDAS) across all time points (4 and 12 weeks) was found.

The analysis of the results should take into account some questions. The sample size was not sufficient to reach an adequate statistical power and we could only speculate on the results. In this sense, the lack of significance in the primary end point in the whole population could be a consequence of a small sample size. However, a clear trend toward a better performance of the onabotulinum toxin A-treated patients across all time points and for all considered parameters, including pain perception and disability measures, was detected. There were no significant differences favoring placebo for any efficacy variable at any time point in the study. Furthermore, the proportion of responders was clearly in favor of onabotulinum toxin A-treated. In addition, as the patients underwent withdrawal treatment due to a medication-overuse, the role of this treatment in the clinical improvement should be taken into account. Withdrawal treatment represents a pivotal strategy to treat patients with MOH [28] and this is also confirmed from the clear clinical improvement observed in MOH patients treated with placebo. However, as the intake of acute pain medication and the withdrawal treatment were similar between the groups (placebo and onabotulinum toxin A), but the proportion of responders was clearly in favor of the onabotulinum toxin A treated, we hypothesized that the injection of onabotulinum toxin A could be responsible for this further significant clinical improvement detectable in MOH patients treated with onabotulinum toxin A.

Another relevant result is the statistically significant improvement in patients-reported quality of life measures, such as HIT-6 and MIDAS scores, observed in onabotulinum toxin A-treated patients with pericranial muscle tenderness and muscular allodynia when compared with the placebo treated.

Treatment-related adverse events were reported in 25.9% of the onabotulinum toxin A-treated (7 patients), and 7.4% of the onabotulinum toxin A-treated patients (2 patients) discontinued due to treatment-related adverse events. No clinically significant serious adverse events were reported in any of the 56 subjects. These data confirm the favorable safety profile of onabotulinum toxin A injected into the head and neck muscles.

Our data confirm and support previous clinical trial findings obtained in patients with chronic migraine in which only two-thirds overused acute pain medication during the baseline period [15–17]. As the presence of medication overuse represents a risk factor for the development of chronification [3, 6] as well as a factor that reduces the efficacy of the prophylactic treatment [8], the success rate of preventing migraine attacks using onabotulinum toxin A in our MOH samples could be considered a further confirmation of the efficacy of this treatment in chronic migraine prophylaxis and, in particular, in patients with peculiar clinical characteristics such as pericranial muscle tenderness.

From a pathophysiological point of view, as in a previous study, we demonstrate that the withdrawal treatment reduces both the clinical severity as well as the sensitization in pain processing that take place in patients with MOH [24]. We hypothesize that, in view of these results, onabotulinum toxin A could influence, through the inhibition of peripheral sensitization [21], the central mechanisms responsible for the facilitation in pain processing, which contribute to the development and maintenance of chronification in these patients.

In conclusion, our data permit the identification as predictor of clinical response to onabotulinum toxin A in patients with complicated form of migraine such as MOH, the presence of pericranial muscle tenderness and so to support it as prophylactic treatment in patients with these features.

## Electronic supplementary material

Below is the link to the electronic supplementary material.

Supplementary material 1 (PDF 348 kb)

Supplementary material 2 (PDF 359 kb)

## References

[1] Headache Classification Subcommittee of the International Headache Society (2004). The international classification of headache disorders: 2nd edition. Cephalalgia.

[2] Headache Classification Committee of the International Headache Society (2006). New appendix criteria open for a broader concept of chronic migraine. Cephalalgia.

[3] Zwart JA, Dyb G, Hagen K, Svebak S, Holmen J (2003). Analgesic use: a predictor of chronic pain and medication overuse headache: the head-HUNT study. Neurology.

[4] Colas R, Munoz P, Temprano R, Gomez C, Pascual J (2004). Chronic daily headache with analgesic overuse: epidemiology and impact on quality of life. Neurology.

[5] Dowson AJ (2003). Analysis of the patients attending a specialist UK headache clinic over a 3-year period. Headache.

[6] Sances G, Ghiotto N, Loi M, Guaschino E, Marchioni E, Catarci T, Nappi G (2005). A CARE: pathway in medication-overuse headache: the experience of the Headache Centre in Pavia. J Headache Pain.

[7] Nappi G, Perrotta A, Rossi P, Sandrini G (2008). Chronic daily headache. Expert Rev Neurother.

[8] Katsarava Z, Muessig M, Dzagnidze A, Fritsche G, Diener HC, Limmroth V (2005). Medication overuse headache: rates and predictors for relapse in a 4-year prospective study. Cephalalgia.

[9] Aurora SK, Gawel M, Brandes JL, Pokta S, VanDenburgh AM (2007). Botulinum toxin type A prophylactic treatment of episodic migraine: a randomized, double-blind, placebo-controlled exploratory study. Headache.

[10] Relja M, Poole AC, Schoenen J, Pascual J, Lei X, Thompson C (2007). A multicentre, double-blind, randomized, placebo-controlled, parallel group study of multiple treatments of botulinum toxin type A (BoNTA) for the prophylaxis of episodic migraine headaches. Cephalalgia.

[11] Schulte-Mattler WJ, Krack P, BoNTTH Study Group (2004). Treatment of chronic tension-type headache with botulinum toxin A: a randomized, double-blind, placebo-controlled multicenter study. Pain.

[12] Padberg M, de Bruijn SF, de Haan RJ, Tavy DL (2004). Treatment of chronic tension-type headache with botulinum toxin: a double-blind, placebo-controlled clinical trial. Cephalalgia.

[13] Silberstein SD, Gobel H, Jensen R (2006). Botulinum toxin type A in the prophylactic treatment of chronic tension-type headache: a multicentre, double-blind, randomized, placebo-controlled, parallel-group study. Cephalalgia.

[14] Aurora SK, Dodick DW, Turkel CC, DeGryse RE, Silberstein SD, Lipton RB, Diener HC, Brin MF (2010). PREEMPT 1 Chronic Migraine Study Group. Onabotulinum toxin A for treatment of chronic migraine: results from the double-blind, randomized, placebo-controlled phase of the PREEMPT 1 trial. Cephalalgia.

[15] Farinelli I, Coloprisco G, De Filippis S, Martelletti P (2006). Long-term benefits of botulinum toxin type A (BOTOX) in chronic daily headache: a five-year long experience. J Headache Pain.

[16] Diener HC, Dodick DW, Aurora SK, Turkel CC, DeGryse RE, Lipton RB, Silberstein SD, Brin MF (2010). PREEMPT 2 Chronic Migraine Study Group. Onabotulinum toxin A for treatment of chronic migraine: results from the double-blind, randomized, placebo-controlled phase of the PREEMPT 2 trial. Cephalalgia.

[17] Dodick DW, Turkel CC, DeGryse RE, Aurora SK, Silberstein SD, Lipton RB, Diener HC, Brin MF (2010). PREEMPT Chronic Migraine Study Group. Onabotulinum toxin A for treatment of chronic migraine: pooled results from the double-blind, randomized, placebo-controlled phases of the PREEMPT clinical program. Headache.

[18] Mathew NT, Kailasam J, Meadors L (2008). Predictors of response to botulinum toxin type A (BoNTA) in chronic daily headache. Headache.

[19] Jakubowski M, McAllister PJ, Bajwa ZH, Ward TN, Smith P, Burstein R (2006). Exploding vs. imploding headache in migraine prophylaxis with botulinum toxin A. Pain.

[20] Burstein R, Dodick D, Silberstein S (2009). Migraine prophylaxis with botulinum toxin A is associated with perception of headache. Toxicon.

[21] Aoki KR (2005). Review of a proposed mechanism for the antinociceptive action of botulinum toxin type A. Neurotoxicology.

[22] Mongini F, Ciccone G, Deregibus A, Ferrero L, Mongini T (2004). Muscle tenderness in different headache types and its relation to anxiety and depression. Pain.

[23] Ayzenberg I, Obermann M, Nyhuis P, Gastpar M, Limmroth V, Diener HC, Kaube H, Katsarava Z (2006). Central sensitization of the trigeminal and somatic nociceptive systems in medication overuse headache mainly involves cerebral supraspinal structures. Cephalalgia.

[24] Perrotta A, Serrao M, Sandrini G, Burstein R, Sances G, Rossi P, Bartolo M, Pierelli F, Nappi G (2010). Sensitisation of spinal cord pain processing in medication overuse headache involves supraspinal pain control. Cephalalgia.

[25] Wolfe F, Smythe HA, Yunus MB, Bennett RM, Bombardier C, Goldenberg DL (1990). The American College of Rheumatology 1990 criteria for the classification of fibromyalgia: report of the Multicenter Criteria Committee. Arthritis Rheum.

[26] Bruehl S, Harden RN, Galer BS, Saltz S, Bertram M, Backonja M (1999). External validation of IASP diagnostic criteria for complex regional pain syndrome and proposed research diagnostic criteria. International Association for the Study of Pain. Pain.

[27] Cruccu G, Anand P, Attal N, Garcia-Larrea L, Haanpää M, Jørum E (2004). EFNS guidelines on neuropathic pain assessment. Eur J Neurol.

[28] Ghiotto N, Sances G, Galli F, Tassorelli C, Guaschino E, Sandrini G, Nappi G (2009). Medication overuse headache and applicability of the ICHD-II diagnostic criteria: 1-year follow-up study (CARE I protocol). Cephalalgia.

[29] Mathew NT, Kailasam J, Seifert T (2004). Clinical recognition of allodynia in migraine. Neurology.

