# Supplementary material for: Botulinum toxin type-A in the prophylactic treatment of medication-overuse headache: a multicenter, double-blind, randomized, placebo-controlled, parallel group study
Source: J Headache Pain. 2011 Apr 16;12(4):427–33. doi: 10.1007/s10194-011-0339-z (PMC3139089; doi:10.1007/s10194-011-0339-z)
Supplement: Supplementary file 1 — Supplementary material 1 (PDF 348 kb) [file 10194_2011_339_MOESM1_ESM.pdf]

Botulinum toxin type-A in the prophylactic treatment of medication-overuse headache: a multicentre, double-blind, randomized, placebo-controlled, parallel-group study.

JOURNAL OF HEADACHE AND PAIN

Giorgio Sandrini; Armando Perrotta*; Cristina Tassorelli; Paola Torelli; Filippo Brighina; Grazia Sances; Giuseppe Nappi

*Corresponding author

Armando Perrotta, MD, PhD

Headache Science Center, IRCCS ‘‘C. Mondino Institute of Neurology’’ Foundation

Via Mondino 2, 27100 Pavia, Italy

Tel.: +39 0382 380435 Fax: +39 0382 380448 E-mail: armando.perrotta@mondino.it

Online Resource 1

Headache pain intensity and assessment of disability (MIDAS and HIT-6) in Medication-Overuse Headache patients at baseline and after Botulinum Toxin Type A (BoNTA) and placebo at 4 and 12 weeks.

		**BoNTA (n=27)**	**Placebo (n=29)**	***p* values**
**Pain intensity (NRS)**	**baseline**	6.8±1.5 (3-10)	7.5±1.2 (5-10)	.100
	**4**	4.2±3.1 (3-9)	5.6±2.7 (0-9)	.110
	**12**	3.6±2.9 (0-8)	5.2±2.6 (0-9)	.099
**MIDAS**	**baseline**	39.0±19.3 (5-79)	52.5±27.7 (12-92)	.142
	**4**	23.3±16.5 (2-60)	40.4±29.0 (6-104)	.070
	**12**	18.0±13.8 (0-58)	33.8±25.5 (6-80)	.064
**HIT-6**	**baseline**	67.7±4.5 (59-74)	68.1±7.1 (52-78)	.649
	**4**	55.9±12.9 (36-76)	61.1±13.2 (36-78)	.187
	**12**	51.3±11.0 (38-70)	58.1±10.2 (36-76)	.093

NRS= numerical rating scale; MIDAS= Migraine Disability Assessment Scale; HIT-6= Headache Impact Test
