# Supplementary material for: Botulinum toxin type-A in the prophylactic treatment of medication-overuse headache: a multicenter, double-blind, randomized, placebo-controlled, parallel group study
Source: J Headache Pain. 2011 Apr 16;12(4):427–33. doi: 10.1007/s10194-011-0339-z (PMC3139089; doi:10.1007/s10194-011-0339-z)
Supplement: Supplementary file 2 — Supplementary material 2 (PDF 359 kb) [file 10194_2011_339_MOESM2_ESM.pdf]

Botulinum toxin type-A in the prophylactic treatment of medication-overuse headache: a multicentre, double-blind, randomized, placebo-controlled, parallel-group study.

JOURNAL OF HEADACHE AND PAIN

Giorgio Sandrini; Armando Perrotta*; Cristina Tassorelli; Paola Torelli; Filippo Brighina; Grazia Sances; Giuseppe Nappi

*Corresponding author
Armando Perrotta, MD, PhD
Headache Science Center, IRCCS ‘‘C. Mondino Institute of Neurology’’ Foundation
Via Mondino 2, 27100 Pavia, Italy
Tel.: +39 0382 380435 Fax: +39 0382 380448 E-mail: armando.perrotta@mondino.it

Online Resource 2

Figure 1,2,3

Mean change (±SE) from baseline in headache pain intensity (Fig.1) and in disability measures Migraine Disability Assessment Scale (MIDAS) (Fig. 2) and Headache Impact Test (HIT)-6 (Fig. 3) at both 4 and 12 week after BoNTA or placebo in MOH patients with pericranial muscle tenderness.

Figure 1

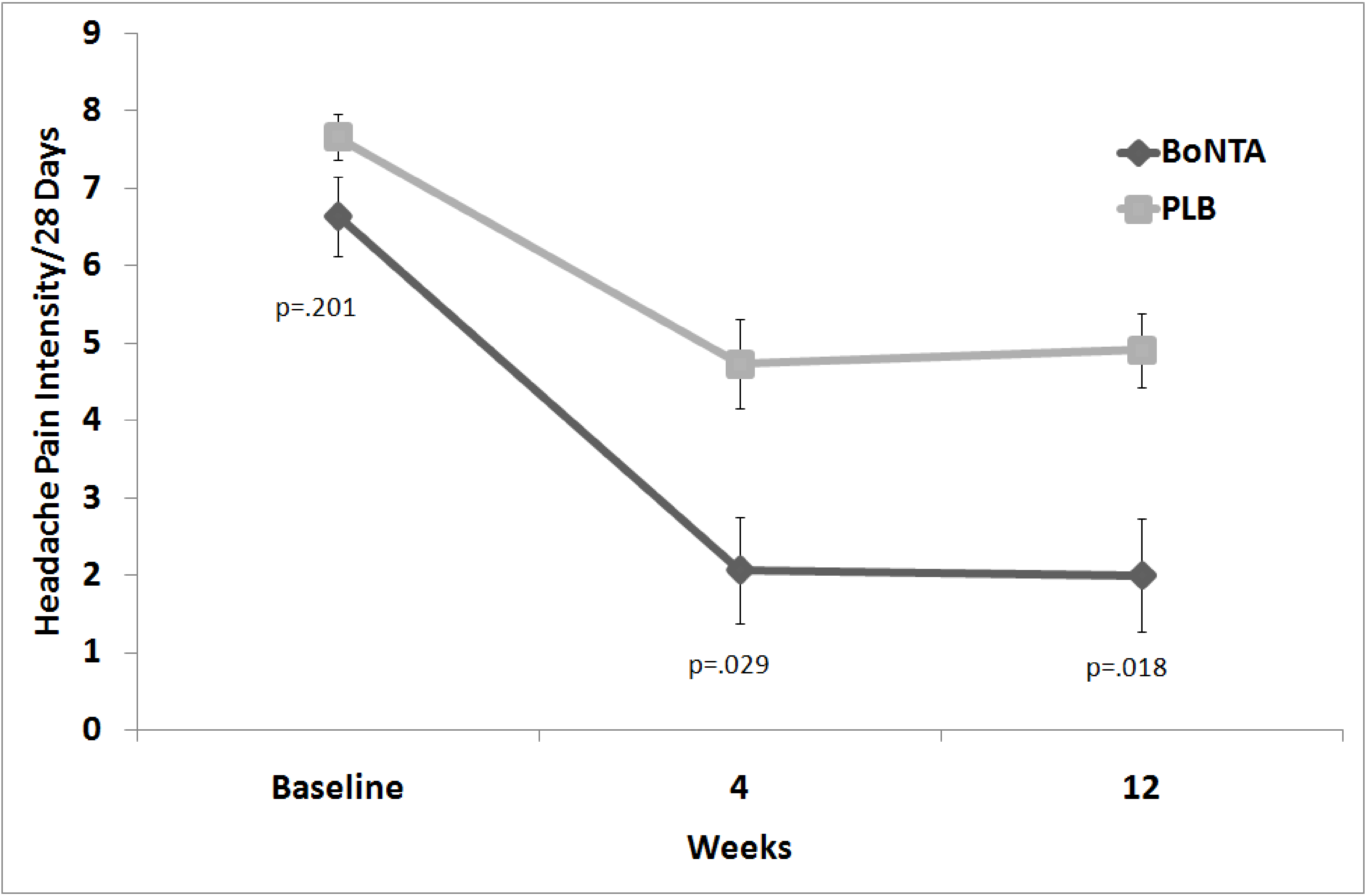

Headache Pain Intensity/28 Days
9
8
7
6
5
4
3
2
1
0
BoNTA
PLB
p=.201
p=.029
p=.018
Baseline
4
12
Weeks

Figure 2

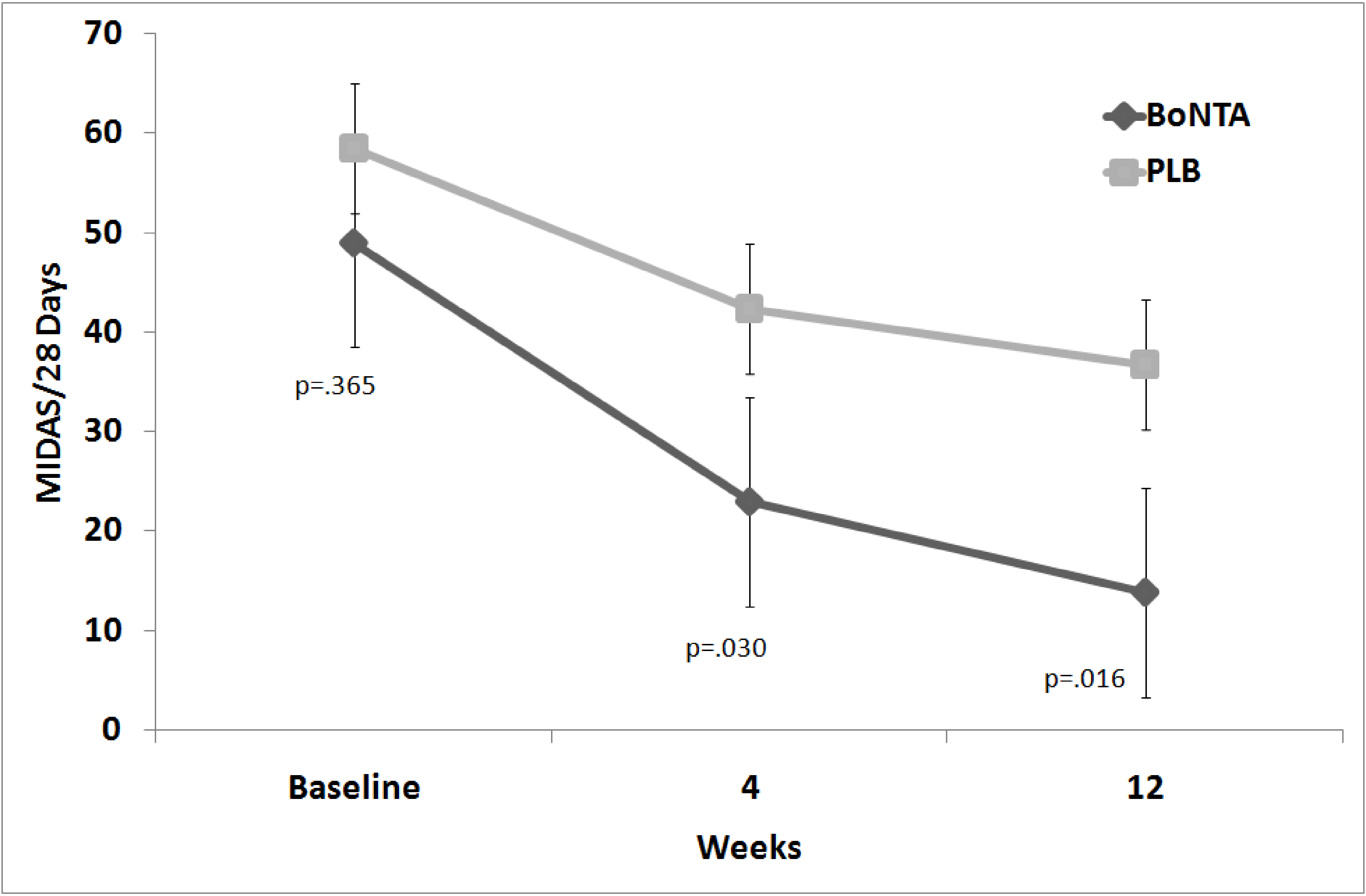
70
60
50
40
30
20
10
0
MIDAS/28 Days
BoNTA
PLB
p=.365
p=.030
p=.016
Baseline
4
12
Weeks

Figure 3

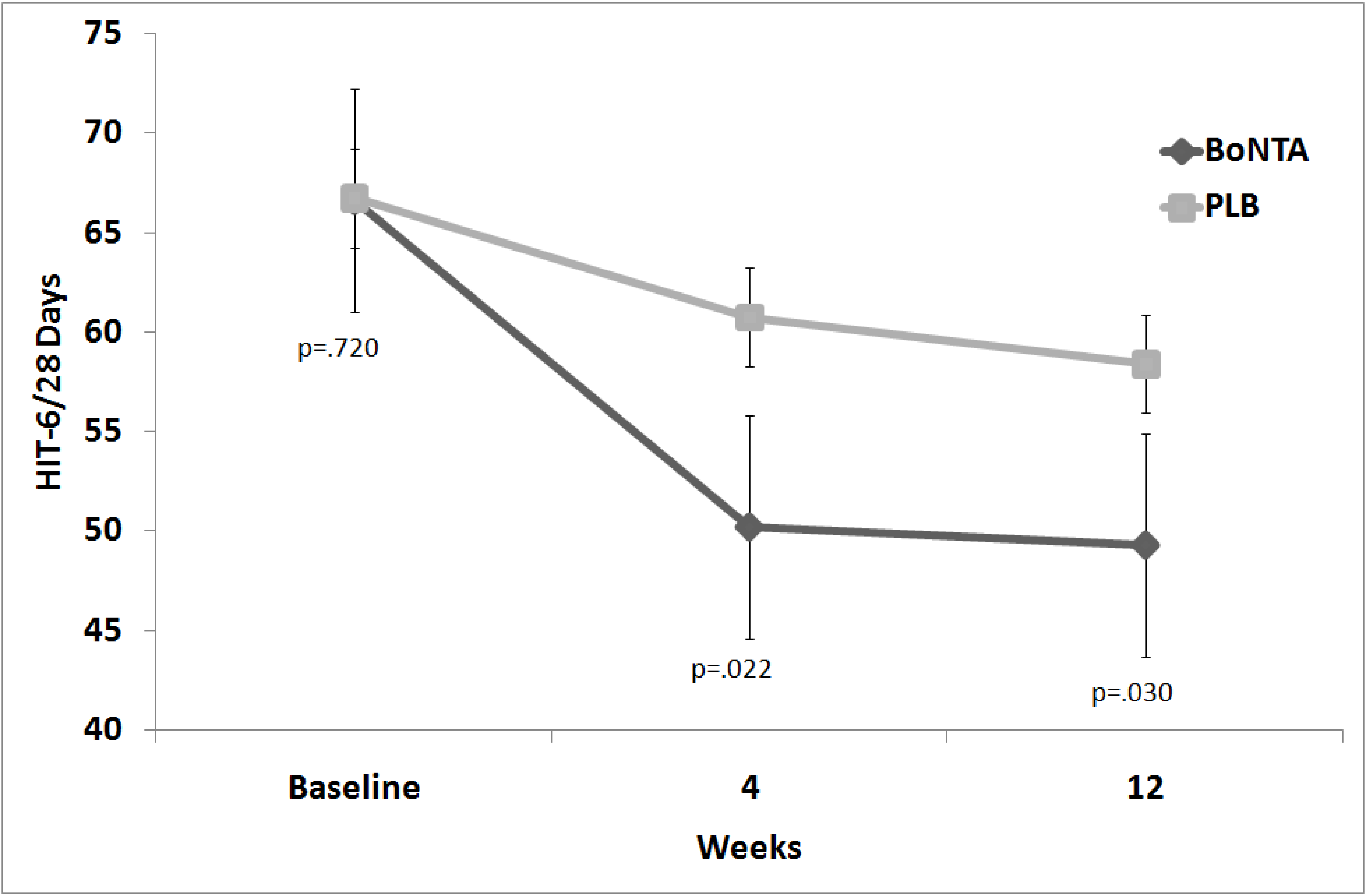

75
70
65
60
55
50
45
40
HIT-6/28 Days
BoNTA
PLB
p=.720
p=.022
p=.030
Baseline
4
12
Weeks
